# Characterization of non-host resistance in broad bean to the wheat stripe rust pathogen

**DOI:** 10.1186/1471-2229-12-96

**Published:** 2012-06-21

**Authors:** Yulin Cheng, Hongchang Zhang, Juanni Yao, Xiaojie Wang, Jinrong Xu, Qingmei Han, Guorong Wei, Lili Huang, Zhensheng Kang

**Affiliations:** 1 State Key Laboratory of Crop Stress Biology for Arid Areas and College of Plant Protection, Northwest A&F University, Yangling, Shaanxi, 712100, People’s Republic of China; 2 College of Life Sciences, Northwest A&F University, Yangling, Shaanxi, 712100, People’s Republic of China; 3 Department of Botany and Plant Pathology, Purdue University, West Lafayette, IN, 47907, USA

**Keywords:** Broad bean, Cell wall, Expression profile, Haustorium, Non-host resistance, Rust fungus

## Abstract

**Background:**

Non-host resistance (NHR) confers plant species immunity against the majority of microbial pathogens and represents the most robust and durable form of plant resistance in nature. As one of the main genera of rust fungi with economic and biological importance, *Puccinia* infects almost all cereals but is unable to cause diseases on legumes. Little is known about the mechanism of this kind of effective defense in legumes to these non-host pathogens.

**Results:**

In this study, the basis of NHR in broad bean (*Vicia faba* L.) against the wheat stripe rust pathogen, *Puccinia striiformis* f. sp. *tritici* (*Pst*), was characterized. No visible symptoms were observed on broad bean leaves inoculated with *Pst*. Microscopic observations showed that successful location of stomata and haustoria formation were significantly reduced in *Pst* infection of broad bean. Attempted infection induced the formation of papillae, cell wall thickening, production of reactive oxygen species, callose deposition and accumulation of phenolic compounds in plant cell walls. The few *Pst* haustoria that did form in broad bean cells were encased in reactive oxygen and callose materials and those cells elicited cell death. Furthermore, a total of seven defense-related genes were identified and found to be up-regulated during the *Pst* infection.

**Conclusions:**

The results indicate that NHR in broad bean against *Pst* results from a continuum of layered defenses, including basic incompatibility, structural and chemical strengthening of cell wall, posthaustorial hypersensitive response and induction of several defense-related genes, demonstrating the multi-layered feature of NHR. This work also provides useful information for further determination of resistance mechanisms in broad bean to rust fungi, especially the adapted important broad bean rust pathogen, *Uromyces viciae-fabae*, because of strong similarity and association between NHR of plants to unadapted pathogens and basal resistance of plants to adapted pathogens.

## Background

Non-host resistance (NHR) is resistance exhibited by an entire plant species to all genetic variants of a non-adapted pathogen species (or bacterial pathovar [pv] or fungal forma specialis [f. sp.]) and represents the most robust and durable form of plant resistance in nature [1]. The presence of this defense system explains why plants are immune to the vast majority of potential pathogens and normally healthy. Molecular mechanisms underpinning NHR remain relatively unexplored compared with the well-studied host resistance mediated by the products of plant resistance (R) genes, which establish pathogen race- or plant cultivar-specific resistance [2,3].

NHR against bacteria, fungi and oomycetes can be divided into two types [4]. Type I NHR does not produce visible symptoms whereas type II NHR results in a rapid hypersensitive response with cell death [4]. Type I NHR is much more common than type II NHR, and NHR of plants against the majority of unadapted pathogens belongs to Type I. Plants have evolved sophisticated mechanisms to exclude unadapted pathogens. An obvious initial requirement for plant disease is basic compatibility where appropriate physical and chemical signals from the plant are required for inducing cell differentiation and expressing essential pathogenicity genes [5,6]. Presence of preformed plant physical and chemical barriers, including plant cell wall and plant surface antimicrobial enzymes and secondary metabolites, are often considered the first line of defense in plants against a pathogen before penetration [6]. Constitutive barriers are more likely to contribute to NHR to pathogens of other plant families than to pathogens of related plant species [7]. After these constitutive barriers are breached, plants have evolved inducible defense mechanisms against invading pathogens. An example of an inducible structural barrier is the formation of papillae. This local cell wall fortification is formed on the inner side of plant cell walls at the penetration site. All microbes possess a suite of conserved molecules, called MAMPs/PAMPs (microbe/pathogen associated molecular patterns) that can be recognized by plants, often via receptor kinase located in the plant plasma membrane [8]. The plant primary innate immune responses are mediated by transmembrane pattern PAMP-triggered immunity (PTI) that can halt further colonization of the pathogen [9]. However, effector triggered immunity (ETI) is not just confined to adapted pathogen recognition and may also play a role in NHR, particularly against pathogens that colonize plant species closely related to non-host species [10].

Obligate biotrophic pathogens, with a specific lifestyle that keeps plant cells alive and minimizes tissue damage in susceptible hosts, are suitable for NHR studies [11]. *Arabidopsis* NHR to non-adapted biotrophic powdery mildews is based upon two successive, multicomponent and independently effective defense systems: *PEN* gene-mediated pre-invasion resistance and *EDS1*/*PAD4/SAG101*-controlled post-invasion immunity [1,12-14]. Compared to powdery mildew fungi, the understanding of NHR mechanisms to rust fungi has lagged behind. *Puccinia* and *Uromyces* represent two large and important genera of rust fungi, which have damaged cereals and legumes, respectively, around the globe throughout history [15]. The emergence of Ug99, a new pathotype of the wheat stem rust pathogen that threatens global wheat production, is a reminder of the need for durable rust resistance in cereals [16,17]. Much effort has been taken to study NHR to rust with non-host pathosystems of *Puccinia*-Gramineae and *Uromyces*-dicotyledons at histological and cytological levels, demonstrating that the majority of rust pathogens are arrested immediately after the formation of the first haustorium mother cell (HMC) in most non-host plant species [18-23]. Several recent studies have investigated the interaction of rust pathogens on non-host plants mainly at molecular levels, including growth of *U. vignae*, *P. triticina*, *Hemileia vastatrix* on *Arabidopsis*[11,24,25], *P. hordei* and *U. fabae* on wheat [26,27], *P. triticina*, *P. hordei-murini*, *P. hordei-secalini* and *P. persistens* on barley [28], and *P. graminis*, *P. triticina*, *P. striiformis*, *P. hordei* and *Melampsora lini* on rice [5,29]. These studies demonstrated that NHR to rust fungi is polygenically inherited and is an active response involving salicylic acid (SA) signaling.

Broad bean (*Vicia faba* L.) is one of the oldest crops cultivated by humans and an important temperate legume crop used as a source of protein in human diets, as fodder and as forage crop for animals, and for available nitrogen in the biosphere [30]. However, broad bean can be seriously damaged by the broad bean rust, caused by *U. viciae-fabae*. Due to the enormous size (13,000 Mbp) and complexity of the broad bean genome [31], research into the functional genomics and cloning of interesting genes has been hampered.

To date, little is known about the nature of effective defense mechanisms in legumes to pathogens of remotely related plant species, especially wheat rust pathogens with economic and biological importance. In this study, NHR in broad bean to the wheat stripe rust pathogen, *P. striiformis* f. sp. *tritici* (*Pst*), was systematically investigated at the histological and molecular levels. The results indicate that NHR in broad bean to *Pst* results from a continuum of layered defenses and provide useful information for further determination of resistance mechanisms of broad bean to rust fungi.

## Results

### Type I NHR to *Pst* on broad bean

**Figure 1:**
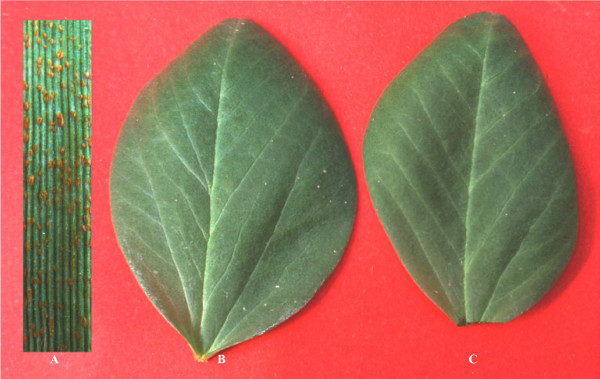
**Leaves of wheat and broad bean inoculated with *Puccinia striiformis *f. sp. *tritici *were checked 14 days post-inoculation. (A)** Massive uredia formed on wheat cultivar Mingxian169. **(B)** No visible symptoms on broad bean cultivar Yuxidabaidou. **(C)** A broad bean leaf mock-inoculated with water.

To determine if different broad bean genotypes show different infection responses to genetically distinct *Pst* races, two broad bean cultivars, Yuxidabaidou and Linxiadacaidou, were inoculated with three Chinese *Pst* races CYR23, CYR31 and CYR32, respectively. No visible symptoms were observed 14 days post-inoculation on broad bean leaves (Figure 1), and similar macroscopic responses were obtained from these different combinations. Thus broad bean displayed type I NHR against *Pst* infection. Additionally, microscopic observations show that no significant differences in fungal growth among these two broad bean cultivars in regard to their interactions with the different *Pst* races (Table 1).

**Table 1:** **No significant differences in fungal growth among two broad bean cultivars in regard to their interactions with three different races of *P *. *striiformis *f. sp. *tritici ***

**Broad bean**	**Linear length of infection hyphae (μm)**		
	**CYR23**	**CYR31**	**CYR32**
Yuxidabaidou ^(1)^	55.47 ± 1.40	54.73 ± 1.21	54.95 ± 1.12
Linxiadacaidou ^(2)^	54.97 ± 0.98	55.07 ± 0.85	54.29 ± 1.23

^(1)^ one main broad bean cultivar grown in Yunnan, China.

^(2)^ one broad bean cultivar grown in Gansu, China.

The linear length of infection hyphae (from the substomatal vesicle to the apex of the longest infection hypha) were measured by microscopy at nine days post-inoculation. Data are mean ± standard deviation, and there is no statistical difference (P > 0.05) compared by Student t tests.

In regard to economic importance, the pathosystem between Yuxidabaidou that is one main broad bean cultivar grown in Yunnan, China, and CYR32, one of two predominant *Pst* races in China, can be taken as a representative of the broad bean-*Pst* non-host interaction and was therefore used for further experiments in the study.

### Growth and development of *Pst* on broad bean

**Figure 2:**
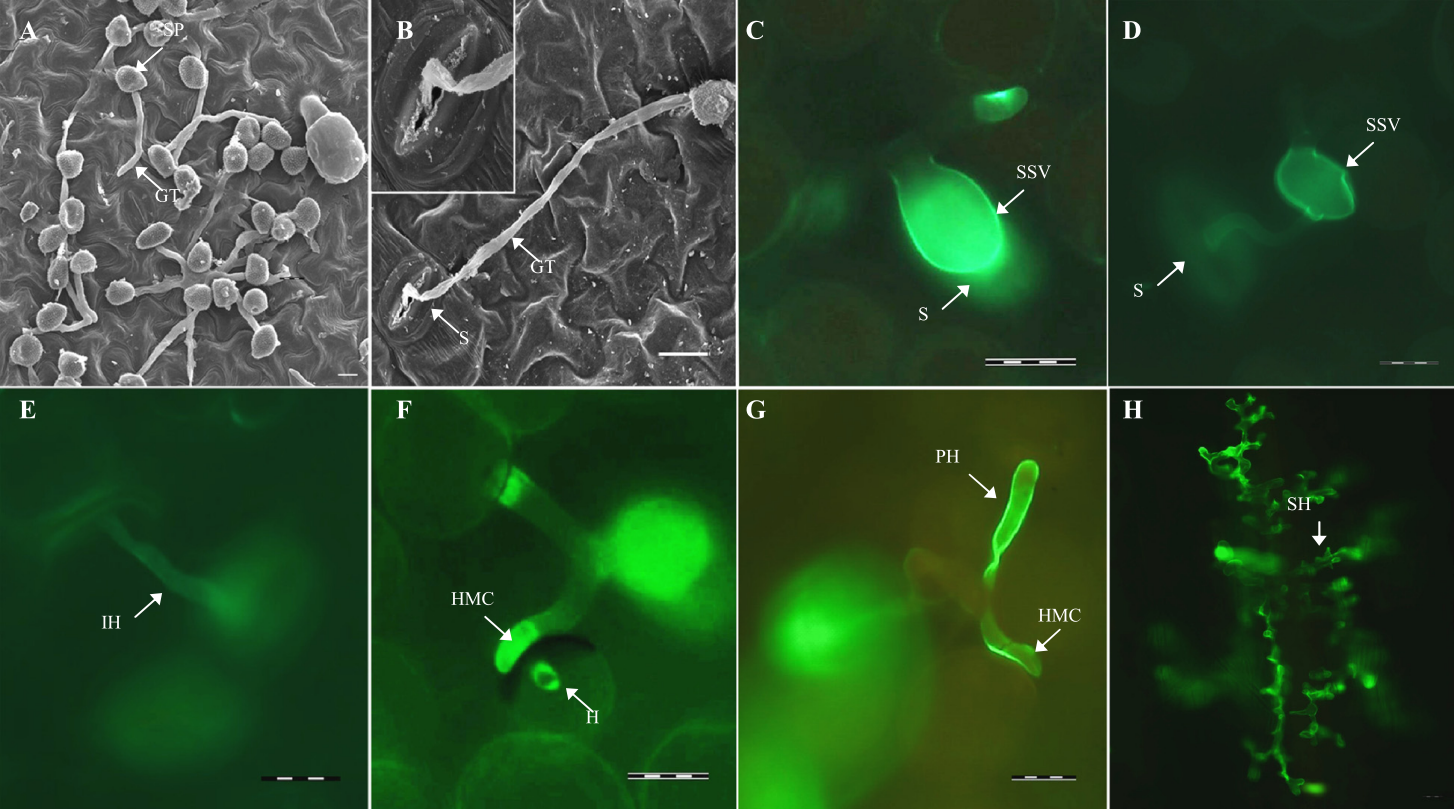
**Infection features of *P. striiformis *f. sp. *tritici *( *Pst *) on broad bean. (A) ** Germinated *Pst * urediniospores (SP) producing germ tubes (GT) growing in random directions at 6 hpi. **(B)** Germ tube (GT) that has successfully located and entered a stomate (S) at 12 hpi. **(C)** A normal oval substomatal vesicle (SSV) formed within the substomatal chamber and adjacent to the stomate (S) at 24 hpi. **(D)** An irregularly shaped SSV formed far from the stomate (S) at 24 hpi. **(E)** Some infection units did not contain a SSV structure and directly produced infection hyphae (IH) at 24 hpi. **(F) ** A *Pst * haustorium (H) within a mesophyll cell after penetration by a haustorial mother cell (HMC) at 24 hpi. **(G, H) ** Broad bean and wheat inoculated with *Pst *, respectively, at 168 hpi. Only one or two primary hyphae (PH) were produced and the vast majority of the *Pst * infection hyphae were blocked at HMC formation in broad bean leaves compared with extensive colonization and formation of secondary hyphae (SH) in wheat leaves. Leaves were examined under a scanning electron microscope **(A, B)** and under an epifluorescence microscope after staining with Calcofluor **(C–E)**, or with WGA-alexa staining **(F–H)**. Bar = 20 μm.

**Figure 3:**
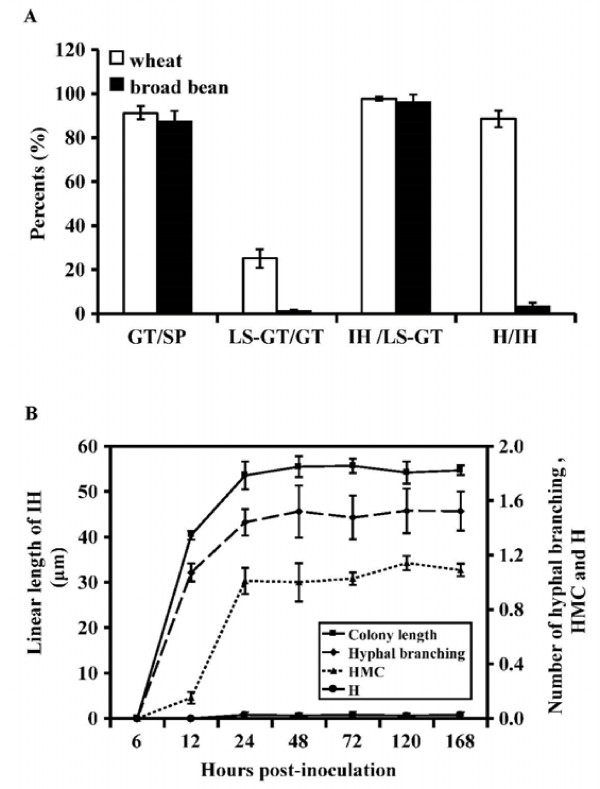
**Limited growth and development of *P. striiformis *f. sp *. tritici *( *Pst *). (A) ** Frequencies of each stage of *Pst * development on broad bean compared with wheat at 48 hpi. SP, urediniospore; GT, germ tube; LS-GT, germ tube locating a stomate; IH, infection hypha; H, haustorium. GT/SP, percentage of the urediniospores producing germ tubes (GT) among total urediniospores (SP); LS-GT/GT, percentage of germ tubes locating stomata (LS-GT) among all germ tubes (GT); IH/LS-GT, percentage of germ tubes locating stomata (LS-GT), successfully penetrating them and forming infection hypha (IH); H/IH, percentage of haustoria (H) formation among all infection hyphae (IH). **(B)** Linear length of infection hyphae (syn. colony length) (from the substomatal vesicle to the apex of the longest infection hypha), and numbers of hyphal branches, haustorium mother cells (HMC) and haustoria (H) formed per infection units measured at continuous time points after inoculation. Linear length of infection hyphae is the left Y-axis; numbers of hyphal branching, haustorium mother cells (HMC) and haustoria (H) are indicated by the right Y-axis. Mean values from three independent replications. Vertical bars represent the standard deviations.

Approximately, 88% of *Pst* urediniospores germinated to produce germ tubes that grew randomly on leaves of the non-host broad bean (Figure 2A, Figure 3A). In a similar fashion, 91% of urediniospores produced germ tubes on wheat (Figure 3A). However, only 1.3% of germ tubes successfully located a broad bean stomate compared to 25% on wheat (Figure 2B, Figure 3A). Of those germ tubes successfully locating a stomate on broad bean, 96% subsequently penetrated it and formed an infection hypha (representing an infection unit) compared with 98% on wheat (Figure 3A). However, of the infection units on broad bean, 28% contained an aberrant substomatal vesicle (SSV) with an irregular oval shape, which did not coincide with stomata (Figure 2D), 22% did not have a SSV structure (Figure 2E), and only 50% contained a normal SSV that formed within the substomatal chamber, which was adjacent to stomata and had a normal oval shape typical of *Pst* on wheat (Figure 2C). A haustorial mother cell (HMC) was formed at the tip of the infection hypha in close contact with mesophyll cells (Figure 2F). However, only 2% of infection hyphae formed haustoria within penetrated mesophyll cells (Figure 2F, Figure 3A). In contrast, 88% of *Pst* infection hyphae produced a haustorium on wheat (Figure 3A). Therefore, successful location of stomata and development of haustoria were greatly reduced in *Pst* infection of broad bean.

The linear length of infection hyphae, and numbers of hyphal branching, haustorial mother cells and haustoria per infection unit at a serial time points post-inoculation were also measured, and they were maintained at approximately 55 μm, 1.5, 1.0 and 0.2, respectively, from 24 hours post-inoculation (hpi) (Figure 3B). Only one or two primary hyphae were produced and the vast majority of the *Pst* infection hyphae were blocked at haustorium mother cell formation in broad bean leaves compared with extensive colonization and formation of secondary hyphae in wheat leaves at 168 hpi (Figure 2G, H). Thus *Pst* development was completely arrested from 24 hpi.

### Histochemical evaluation of broad bean NHR response to *Pst*

**Figure 4:**
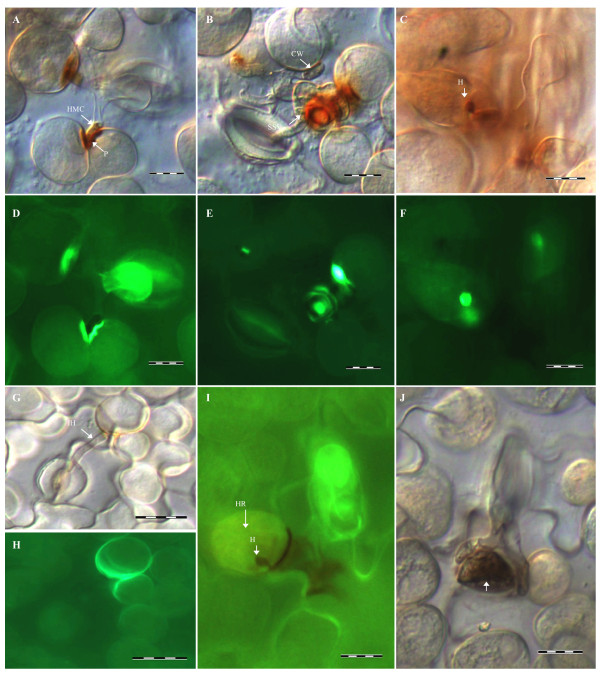
**Broad bean responses to attempted *P. striiformis *f. sp *. tritici *( *Pst *) infection. (A–C)** Inoculated broad bean leaves were stained with 3,3-diaminobenzidine (DAB) to detect production of H_2_O_2_ (reddish brown) at 24 hpi. A dome-shaped papilla (P) **(A)** and thickened cell walls (CW) **(B)** were formed in the broad bean cell walls in contact with the fungal haustorial mother cells (HMC). DAB staining was visible in the mesophyll cell wall adjacent to a HMC **(A)**, the mesophyll and epidermal cell in contact with a substomatal vesicle (SSV) **(B)**, the papilla **(A)**, the encasement surrounding the haustorium (H) **(C) **, and an HMC of *Pst ***(A)**. **(D–F)** Callose deposition (intensive green fluorescent) coinciding with H_2_O_2_ distribution **(A–C)** was detected by aniline blue staining at 24 hpi. **(G)** A mesophyll cell in contanct with an infection hypha (IH) at 24 hpi. **(H)** Bright fluorescence was observed in the cell walls of the mesophyll cell and a neighboring mesophyll cell at the same infection unit shown in **(G)**. **(I)** In some infection units with formation of haustoria (H), plant cell autofluorescence, indicative of HR was detected at 24 hpi. **(J)** Collapsed mesophyll cell (arrow) at 72 hpi. Bar = 20 μm.

**Figure 5:**
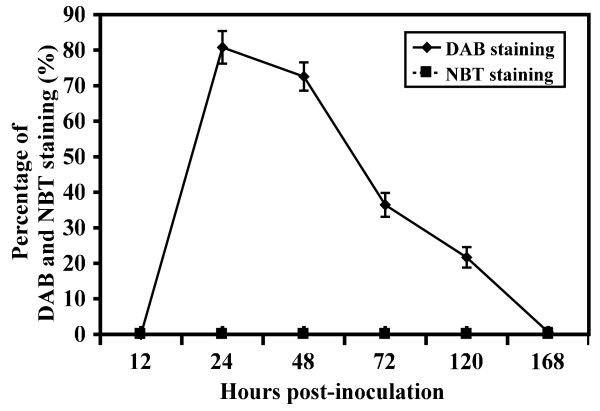
**Percentage of infection units exhibiting H**_**2**_**O**_**2**_**(DAB staining) and O**_**2**_^**-**^**(NBT staining) accumulation on broad bean after inoculation with *P *. *striiformis *f. sp. *tritici *.** Each point represents at least 50 infection units from eight to ten inoculated broad bean leaves. These experiments were repeated three times with similar results. Bars represent standard deviation.

An active NHR response was shown to be involved in the suppression of *Pst* growth on broad bean. A dome-shaped papilla was formed on the inner side of broad bean cell walls at the penetration site adjacent to the HMC (Figure 4A, D). Thickened cell walls were also present in broad bean mesophyll cells in contact with HMC (Figure 4B). The production of H_2_O_2_ and O_2_^-^ in broad bean against *Pst* was analyzed by staining with 3,3^′^-diaminobenzidine (DAB) and nitroblue tetrazolium (NBT), respectively. Accumulation of H_2_O_2_ was detected at the sites in direct contact with SSV or HMC, and in papilla (Figure 4A, B). In infection units with haustorium formation, DAB staining was detected at deposits responsible for haustorium encasement (Figure 4C). DAB staining was occasionally observed in the HMC (Figure 4A). The percentage of infection units with DAB staining increased rapidly from 12 to 24 hpi and then gradually declined (Figure 5). NBT staining was not detected in plant cells of any sample (Figure 5).

Aniline blue staining indicated that callose deposition was associated with the rust infection units and staining at 24 hpi showed the same distribution as H_2_O_2_ in the plant cell walls at the sites in direct contact with SSV or HMC, in papilla, and at encasement of haustoria (Figure 4D-F). Therefore, attempted *Pst* infection of broad bean resulted in rapid production and accumulation of H_2_O_2_ (but not O_2_^-^) and callose deposition, presumably related to the generation of subcellular, localized physical barriers.

The cell walls of mesophyll cells in contact with infection hyphae and neighboring mesophyll cells showed intense bright fluorescence (Figure 4G, H), which is likely related to the accumulation of antimicrobial phenolic compounds. Hypersensitive response (HR) with cell death occurred in infection units with haustoria formation as evidenced by plant cell autofluorescence at 24 hpi (Figure 4I). Penetrated mesophyll cells began to collapse from 48 hpi (Figure 4J).

Collectively, attempted infection induced the formation of papillae, cell wall thickening, production of reactive oxygen species, callose deposition, and accumulation of phenolic compounds in non-host plant cells. The few haustoria that were formed by *Pst* in broad bean cells were encased in reactive oxygen and callose materials and elicited a posthaustorial hypersensitive response.

### Quantitative real-time PCR (qRT-PCR) of seven candidate defense-related genes

Seven defense-related genes that may be involved in basal resistance, oxidative stress responses and callose formation were selected for qRT-PCR assays. They are putative pathogenesis-related gene 1 (*PR1*), pathogenesis-related gene 2 (*PR2*), pathogenesis-related gene 5 (*PR5*), pathogenesis-related gene 10 (*PR10*), superoxide dismutase (*SOD*), catalase (*CAT*), glucan synthase-like 5 (*GSL5*). These genes on broad bean were identified using homologous sequences deposited in GenBank of species closely related to broad bean (Table 2) for designing a set of primers (see Additional file 1). These genes [GenBank: JQ043345-JQ043351] showed 75–97% identities to those used to design primers, and were used for assessments of expression by qRT-PCR, although their full lengths were not available.

**Table 2:** Descriptions of candidate defense-related genes

**Name**	**Annotation**	**Related species**	**Accession number**^**(1)**^	**Arabidopsis homolog locus**^**(2)**^	**BLASTX score/E Value**^**(3)**^
	**Basal resistance**				
*PR1*	Pathogenesis-related gene 1	*Pisum sativum*	CAE51954.1	AT2G14610.1	200/4e-52
*PR2*	Beta-1, 3-endoglucanase	*Pisum sativum*	AAB24398.1	AT3G57260.1	242/5e-80
*PR5*	Thaumatin-like protein	*Medicago truncatula*	TC100682 ^(4)^	—	—
*PR10*	Pathogenesis-related gene 10	*Pisum sativum*	U31669.1	— ^(5)^	**—**
	**Oxidative stress responses**				
*SOD*	Superoxide dismutase	*Pisum sativum*	CAA39819.1	AT5G18100.1	181/5e-61
*CAT*	Catalase	*Pisum sativum*	BAH37035.1	AT1G20620.1	828/0
	**Papillary callose formation**				
*GSL5*	Glucan synthase-Like 5	*Medicago truncatula*	ABN09771.1	AT4G03550.1	1110/0

^(1)^ GenBank accession number: www. ncbi.nlm.nih.gov.

^(2)^ Closest Arabidopsis homolog identified using TAIR BLAST.

^(3)^ score/E value between Arabidopsis homolog locus and legume species by BLASTX.

^(4)^Medicago truncatula Gene Index tentative consensus (TC) numbers for cDNAs on the microarray (http://www.tigr.org/tigr-scripts/tgi/T_index.cgi?species=medicago).

^(5)^ No sequence of *PR10* in *Arabidopsis* using TAIR BLAST.

**Figure 6:**
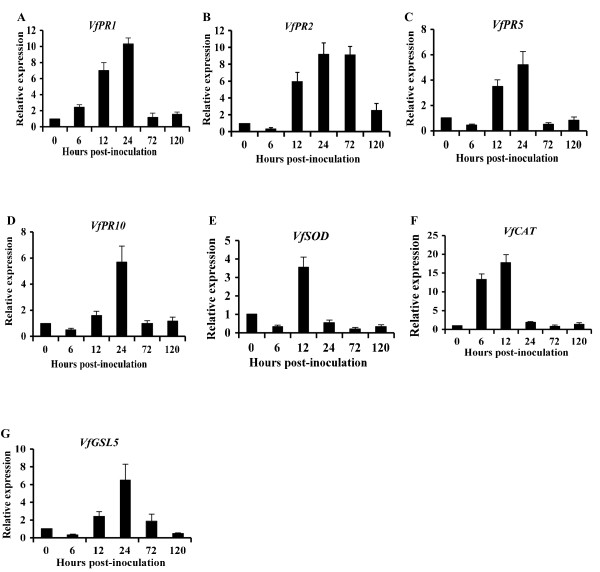
**Relative transcript levels of seven defense-related genes assayed by qRT-PCR.** Relative gene expression was quantified by the comparative 2^-△△CT^ method. The mean expression value was calculated from three independent replications. Vertical bars represent the standard deviations.

A set of primers based on the cloned sequences of the seven defense-related genes were designed (see Additional file 2), and qRT-PCR was performed to test their expression profiles during *Pst* infection across a series of time points post-inoculation on broad bean. As shown in Figure 6, accumulations of *VfPR1*, *VfPR2*, *VfPR5*, and *VfPR10* transcripts were up-regulated as early as 12 hpi, peaked at 24 hpi, and declined to original expression levels except for *VfPR2* that remained at a high level at 72 hpi (Figure 6A-6D). Accumulations of transcripts of *VfSOD* and *VfCAT*, which are involved in oxidative stress peaked at 12 hpi, but the induction of *VfCAT* expression was as early as 6 hpi (Figure 6E, F). Transcription of *VfGSL5*, a callose formation gene, was up-regulated from 12 hpi to 24 hpi and then sharply declined (Figure 6G). These results indicated that *Pst* infection triggered the induction of a set of defense-related genes in broad bean peaking at 12 and 24 hpi.

## Discussion

Non-host resistance (NHR) with obvious durability has been studied as a source of resistance traits that might help in improving crop performance in the glasshouse and the field [6,32]. In the present study, a continuum of layered defenses led to NHR in broad bean. No visible symptoms were observed on broad bean leaves inoculated with *Pst*, indicating type I NHR. However, a hypersensitive response (HR) with cell death in infection units with haustorium formation was also observed in broad bean leaves by microscopic examination. Similar results were observed in the interactions between other rust fungi and non-host plants [20,22,23]. In NHR analysis of *Arabidopsis* to *Phytophthora infestans* infection, penetrated epidermal cells also displayed a rapid hypersensitive response, although no symptoms could be detected [33]. Therefore, HR that cannot be detected on visual symptoms, may be observed at the single cell level in Type I NHR, which is probably related to low infection probability and an extremely rapid HR on non-host plants.

On non-host broad bean leaves, urediniospores of *Pst* germinated at a similar frequency to those on wheat leaves. However, the germ tubes grew randomly without directional growth on broad bean leaves. There was an approximate twenty fold difference in successful location of stomata on broad bean compared to wheat. It appeared that urediniospores germinated well but had difficulty in locating and recognizing stomata on non-host plants [20]. This difference may be due to inappropriate thigmotrophic or biochemical signals arising from the broad bean leaves. This basic incompatibility therefore reduced *Pst* infection efficiency on broad bean by reducing the probability of germ tubes locating stomata (25% on wheat compared with 1.3% on broad bean). This was somewhat comparable to 85% and 12% reported for *P. triticina* (*Pt*) on its wheat host compared to a non-host, *Arabidopsis thaliana*[24]. Moreover, in a graminaceous comparison similar inefficiencies by *Pst*, *Pt,* and *P. graminis* f. sp *tritici* (*Pgt*) in locating stomata in rice compared to wheat were observed (unpublished results)*.* Again *Pst* was more inefficient than the other two species. The major difference in the infection process between *Pst* and other rust fungi is that it does not form obvious appressoria, and germ tubes directly penetrate the stomata. This possibly increases the difficulty in locating and recognizing stomata.

Among *Pst* infection units, some contained an aberrant substomatal vesicle (SSV), and some did not. This difference may be related to different leaf structures between legume and cereal plants, especially the structure and size of the substomatal chambers. The vast majority of *Pst* infection hyphae were blocked at haustorium mother cell formation, which is likely due to plant resistance responses, including papilla formation and cell wall thickening. In other incompatible leguminous-rust or powdery mildew interactions, papilla were frequently observed on plant cell walls [34-36]. It is possible that papilla formation is one of general defense responses effective against biotrophic pathogens in legumes. Attempted *Pst* infection also induced the production of reactive oxygen species, callose deposition, and the accumulation of phenolic compounds in plant cells. We also observed that the haustoria were encased in reactive oxygen and callose materials and elicited plant cell death. Hydrogen peroxide (H_2_O_2_) and callose deposition with the same distribution were detected at 24 hpi, coinciding with the fact that the activation of *CAT*, *SOD* and *GSL5* genes at 12 to 24 hpi. The balance between *SOD* and *APX* or *CAT* activities in cells is crucial for determining a steady-state balance of superoxide radicals and hydrogen peroxide [37]. *U. fabae* infection also triggered the induction *SOD* and *CAT* genes in wheat [26]. *GSL5* encodes a glucan synthase involved in papillary callose formation and callose encasement of haustorial complexes in response to the powdery mildew pathogen [38]. *GSL5* gene was also activated in *Arabidopsis thaliana* against the coffee leaf rust fungus *Hemileia vastatrix*[25]. *GSL5* may therefore be also involved in papillary callose formation and callose encasement of haustorial complexes in response to the rust fungi. H_2_O_2_ can facilitate cross-linking of cell walls [39], and callose (β-1,3-glucan) deposition may reinforce cell walls at contact sites with fungal structures. Phenolic compounds, as one of chemical barrier in inducible defense mechanisms in Type I NHR [4], have been reported to have antimicrobial activity [40].

Therefore, prehaustorial NHR with basic incompatibility and structural and chemical strengthening of cell walls against the majority of penetrating fungal units, and posthaustorial NHR with a hypersensitive response against the few successful penetrations, collectively contribute to the NHR of broad bean to *Pst*. These features also demonstrate the multi-layered feature of NHR.

Pathogen-related proteins encompass several different groups of structurally and functionally unrelated proteins with antimicrobial activities [41]. Four PR genes observed were up-regulated in response to *Pst* infection in broad bean leaves. The expression of all four PR genes is SA responsive [42,43]. *PR1* is widely thought to be a molecular marker for the SA-dependent resistance signaling pathway [41,44]. Thus, SA-mediated resistance pathway is involved in the non-host broad bean-*Pst* interaction. *PR2* protein, which has β-1,3-glucanase activity, can degrade fungal cell walls, together with *PR3* protein, which has chitinolytic activity, cause the lysis of fungal cell walls [45]. We failed to isolate the *PR3* gene from broad bean. Nevertheless, transcription of the *PR2* gene occurred at a high level from 12 to 72 hpi, likely playing a role in inhibiting the growth of *Pst*. Plasma membrane- permeabilizing ability proper to *PR5* contributes to plasmolysis and damage of fungal and bacterial pathogens, inhibiting their growth and development [46]. *PR10* involves in plant defense responses and has antimicrobial activity and in vitro ribonuclease activity [47,48].

In the non-host interaction between broad bean and *Pst*, transcript accumulations of PR genes and the *GSL5* gene involved in callose formation were up-regulated at 12–24 hpi with a peak at 24 hpi, whereas transcript accumulations of *SOD* and *CAT* involved in oxidative stress responses were up-regulated as early as 6–12 hpi with peaks at 12 hpi. The accumulation of hydrogen peroxide (H_2_O_2_) can lead to SA synthesis [49], and elevated levels of SA along with H_2_O_2_ can activate local PR gene expression [50]. H_2_O_2_ produced during early plant-pathogen interactions also contributes to cell wall strengthening processes, such as the formation of papilla where callose is abundantly deposited [51]. Thus, H_2_O_2_ as a singal substance may activate the expression of PR genes and contribute to callose formation in the non-host interaction. This could explain the earlier induction of *SOD* and *CAT* compared to PR genes and *GSL5*.

Although limited by current genomic and transcriptomic data for broad bean, several defense-related genes were successfully identified using the homology cloning approach. We failed to get specific amplification products when using primers designed on the basis of soybean (*Glycine max*) gene sequences. In contrast, positive amplification was obtained using primers designed from pea (*Pisum sativum*) sequences. Similar results were obtained with amplification of other genes [52]. Because of huge genome in broad bean, it was necessary to rely on synteny with related species in order to identify interesting genes [52]. This approach allowed us to identify candidate genes responsible for agronomically important traits in broad bean.

## Conclusions

In this study, we characterized the basis of NHR in broad bean against *Pst*, a pathogen of remotely related plant species, at the histological and molecular levels. Our results indicate that the NHR involves a continuum of layered defense processes, including basic incompatibility, structural and chemical strengthening of the cell wall, posthaustorial hypersensitive response and induction of several defense-related genes, demonstrating the multi-levels feature of NHR. NHR of plants to unadapted pathogens exhibits strong similarity and association with basal resistance of plants to adapted pathogens [7]. Therefore, this study of NHR to *Pst* in broad bean provides useful information for further determination of resistance mechanisms in broad bean to rust fungi, especially the adapted important broad bean rust pathogen, *U. viciae-fabae*. Meanwhile, we confirm that HR can be observed at the single cell level, although there are no visible symptoms in Type I NHR.

## Methods

### Plants, pathogens and inoculation

Two broad bean cultivars used in this study are Yuxidabaidou and Linxiadacaidou. Yuxidabaidou is one main cultivar grown in Yunnan (the main growing area for broad bean in China) and Linxiadacaidou is one cultivar grown in Gansu, China. Wheat cultivar Mingxian169 and *Pst* races CYR23, CYR31, CYR32 used in this study were obtained from the College of Plant Protection. Plants were grown in a soil mixture in 10 cm diameter pots in a growth chamber at 20°C with 60% relative humidity and a 16 h photoperiod (60 μmol m^−2^ s^−1^ photon flux density). Broad bean plants at the 5–6 leaf stage and wheat at the 1–2 leaf stage were used for inoculation. *Pst* was maintained and propagated on the susceptible wheat cultivar Mingxian 169.

For inoculation, fresh urediniospore suspensions (50 mg ml^−1^) were applied with a fine paintbrush onto the adaxial surfaces of leaves of broad bean and wheat plants. Parallel mock inoculations were performed with tap water. Inoculated seedlings were put into a dark humidity chamber for 24 h at 16°C and then moved to the growth chamber. Leaf samples were collected at specific time points for various analyses and disease symptoms were recorded 14 dpi.

### Light microscopy

Infected broad bean and wheat leaf pieces of 2–3 cm^2^ were harvested at set time points, fixed and decolorized in ethanol/trichloromethane (3:1, v/v) containing 0.15% (w/v) trichloroacetic acid for 3–5 days and then cleared in saturated chloral hydrate until leaf tissues were translucent. For microscopic observations, leaf segments were stored in 50% glycerol and examined under differential interference contrast (DIC) optics.

To visualise the pathogen structures, the leaves were stained with Calcofluor (Sigma-Aldrich, St. Louis, MO, USA) [53]. For better visualisation of internal infection structures, the staining procedure for wheat germ agglutinin (WGA) conjugated to the fluorophore alexa 488 (Invitrogen, USA) was also used as described previously [29]. All fluorescence stained tissues were examined under a fluorescence microscope.

For each broad bean leaf sample, at least 50 infection units from 8–10 leaf segments were examined for recording linear length of infection hyphae, and numbers of hyphal branching, haustorial mother cells and haustoria per infection unit. The linear length of infection hyphae was measured from the substomatal vesicle to the apex of the longest infection hypha. All microscopic examinations were done with an Olympus BX-51 microscope (Olympus Corporation, Japan).

### Scanning electron microscopy

Leaf tissues were cut into small pieces (0.5–1 cm), fixed in 4% (v/v) glutaraldehyde in phosphate buffer (0.1 M, pH 6.8) for 8–10 h at 4°C, then rinsed with the same buffer for 1–2 h. After dehydration in a graded ethanol series, the samples were critical-point dried, coated with gold in a sputter coater, and examined under a JEOL JSM-6360 LV scanning electron microscope at 15 kV.

### Histochemical analysis

H_2_O_2_ and O_2_^-^ production was detected in plant tissue by staining with 3-3′ diaminobe-nzidine (DAB) (Amresco, Solon, OH, USA) and nitroblue tetrazolium (NBT) (Amresco, Solon, OH, USA), respectively. Specimen preparation and microscopic observations were performed following procedures previously described [54,55].

Callose deposition was visualised under UV light after modified aniline blue staining as described previously [56]. After DAB uptake, leaf segments were fixed and cleared, and washed twice with 50% (v/v) ethanol for 15 min, rinsed with water, then incubated in 0.067 M K_2_HPO_4_ (pH 9.0) for 30 min, and stained with 0.05% (w/v) aniline blue overnight. Specimens were examined with an Olympus BX-51 microscope (Olympus Corporation). Autofluorescence of attacked mesophyll cells was observed as necrotic cell death by an epifluorescence microscopy (excitation filter, 485 nm; dichromic mirror, 510 nm; and barrier filter, 520 nm).

### RNA extraction and cDNA synthesis

Total RNA of broad bean leaves was extracted using the TrizolTM Reagent (Invitrogen, Carlsbad, CA, U.S.A.). DNaseI treatment was applied to remove genomic DNA. The integrity of total RNA was checked by formamide denaturing gel electrophoresis, and the concentration was determined with a NanoDropTM 1000 spectrophotometer (Thermo Fisher Scientific, U.S.A.). About 3 ug total RNA was used to generate the first-strand cDNA using the Promega RT-PCR system (Promega, Madison, WI, U.S.A.) with the Oligo (dT)18 primer and cDNA constructions were carried out according to the manufacturer’s instructions.

### Identification of defense-related genes

Each gene were firsted identified from *Arabidopsis* and used in BLASTX analysis to search for corresponding orthologous sequences in legume species closely related to broad bean (Table 2). Then, gene-specific primers at conservative positions were designed with Primer Premier 5.0 and used in RT-PCR for cloning the targeted broad bean genes (see Additional file 1). PCR products were cloned and sequenced, followed by analysis using the BLASTX algorithm to confirm the gene specificity.

### Quantitative real-time PCR analysis

Gene expression patterns of seven genes were analyzed by qRT-PCR analysis. Based on the broad bean genes identified, primers were designed ( Additional file 2). Eukaryotic elongation factor 1-alpha (ELF1A) [GenBank: O24534] was used as an internal reference for the qRT-PCR analysis [52]. Quantification of gene expression was performed using a 7500 Real-Time PCR System (Applied Biosystems, Foster City, CA, USA). To avoid variations caused by experimental conditions, the expression level of each gene in the mock-inoculated control was subtracted from that in broad bean leaves inoculated with *Pst.* Dissociation curves were generated for each reaction to ensure specific amplification. Threshold values (CT) generated from the ABI PRISM 7500 Software Tool (Applied Biosystems, Foster City, CA, USA) were used to quantify relative gene expression using the comparative 2^-△△CT^ method [57].

### Statistical analyses

All experiments were repeated three times with similar results. At least 50 infection sites from eight to ten leaf sections per time point were examined in histopathological and histochemical sections. Means and standard deviation were estimated from three independent experiments using SAS software.

## Abbreviations

DAB: 3,3-diaminobenzidine; DIC: Differential interference contrast; ETI: Effector triggered immunity; HMC: Haustorial mother cell; hpi: Hours post-inoculation; HR: Hypersensitive response; MAMPS or PAMPs: Microbial- or pathogen-associated molecular patterns; NBT: Nitroblue tetrazolium; NHR: Non-host resistance; *Pst*: *Puccinia striiformis* f. sp. *tritici*; PTI: PAMP-triggered immunity; qRT-PCR: Quantitative realtime-PCR; SA: Salicylic acid; SSV: Substomatal vesicle.

## Competing interests

The authors declare that they have no competing interests.

## Authors’ contributions

YLC designed experiments, performed the experiments and wrote manuscript. HCZ designed experiments and provided assistance in some experiments. JNY performed cytological experiments and provided assistance in some experiments. XJW and JRX provided advice for experiments and revised the manuscript. GRW prepared samples and collected data. LLH coordinated the experiments and analyzed the data. ZSK conceived the project, helped design the experiments and wrote the manuscript. All authors read and approved the final manuscript.

## Supplementary Material

Additional file 1 Primers used for cloning the seven defense-related genes. Click here for file

Additional file 2 Primers used in quantitative real-time PCR. Click here for file
