# Supplementary material for: Characterization of non-host resistance in broad bean to the wheat stripe rust pathogen
Source: BMC Plant Biol. 2012 Jun 21;12:96. doi: 10.1186/1471-2229-12-96 (PMC3487988; doi:10.1186/1471-2229-12-96)
Supplement: Additional file 1 — Primers used for cloning the seven defense-related genes. [file 1471-2229-12-96-S1.doc]

**Additional file 1：Primers used for cloning the seven defense-related genes.**

| Gene | Forward primer sequence (5'–3') | Reverse primer sequence (5'–3') |
| --- | --- | --- |
| *PR1* | AAACAACATCCATCAAAACCA | ATTACCAGGTGGATCATAGTTACA |
| *PR2* | ATGATGGGAAACAACCTACC | TTACATATCACTCTTAAGAGAAACAG |
| *PR5* | TGCCTTAGCTTTGCATTCC | AAAGTGCTGCTCTTATCATCG |
| *PR10* | ATGCTGATAACCTTACTCCAAA | TTAGTTGTAATCAGGATGAGCCA |
| *SOD* | ATGGCTTCACAAACTCTCGTC | TTATACTGGAGTCAAGCCAACC |
| *CAT* | ATGGATCCTTACAAGCATCGT | TTAAATGCTAGGCCTCATGTTC |
| *GSL5* | AAGAAGCCTTGAAAATGAGAAA | GAACGAATGACTGAGAAGCAAG |
