# Supplementary material for: Characterization of non-host resistance in broad bean to the wheat stripe rust pathogen
Source: BMC Plant Biol. 2012 Jun 21;12:96. doi: 10.1186/1471-2229-12-96 (PMC3487988; doi:10.1186/1471-2229-12-96)
Supplement: Additional file 2 — Primers used in quantitative real-time PCR. [file 1471-2229-12-96-S2.doc]

| Gene | Forward primer sequence (5'–3') | Reverse primer sequence (5'–3') |
| --- | --- | --- |
| *VfPR1* | CAGTGGTGACATAACAGGAGCAG | CATCCAACCCGAACCGAAT |
| *VfPR2* | CCAATGGGTACAAAGAAACG | AAACCAAGTAACCAATGAAAGG |
| *VfPR5* | TCTGTAACTCCACAAGGCGG | TGTATTATGACTTCCACGGCAA |
| *VfPR10* | GCAGGACCAAATGGAGGAT | TCAGGATGAGCCAAACAGTAAC |
| *VfSOD* | CTGCCGCCAAGAAAGCC | GGTCCTGTTGAGATACACCCATT |
| *VfCAT* | GATTTTGACCCACTTGATGTAACC | AGGCACGATAATGGCAGGAC |
| *VfGSL5* | CCTGATGTCTTTGATAGGATTTTTC | TCTCTTCCCTTCCCAACTTGTA |
| *VfELF1A* | GTGAAGCCCGGTATGCTTGT | CTTGAGATCCTTGACTGCAACATT |

**Additional file 2：Primers used in quantitative real-time PCR.**
